# *Sarve*: synthetic data and local differential privacy for private frequency estimation

**DOI:** 10.1186/s42400-022-00129-6

**Published:** 2022-08-03

**Authors:** Gatha Varma, Ritu Chauhan, Dhananjay Singh

**Affiliations:** 1 Amity Institute of Information Technology, Amity University, Noida, India; 2 Center for Computational Biology and Bioinformatics, Amity University, Noida, India; 3 Department of Electronics Engineering, HUFS, Seoul, Korea

**Keywords:** Synthetic data, Differential privacy, Frequency estimation, Frequency oracle, Privacy

## Abstract

The collection of user attributes by service providers is a double-edged sword. They are instrumental in driving statistical analysis to train more accurate predictive models like recommenders. The analysis of the collected user data includes frequency estimation for categorical attributes. Nonetheless, the users deserve privacy guarantees against inadvertent identity disclosures. Therefore algorithms called frequency oracles were developed to randomize or perturb user attributes and estimate the frequencies of their values. We propose *Sarve*, a frequency oracle that used Randomized Aggregatable Privacy-Preserving Ordinal Response (RAPPOR) and Hadamard Response (HR) for randomization in combination with fake data. The design of a service-oriented architecture must consider two types of complexities, namely computational and communication. The functions of such systems aim to minimize the two complexities and therefore, the choice of privacy-enhancing methods must be a calculated decision. The variant of RAPPOR we had used was realized through bloom filters. A bloom filter is a memory-efficient data structure that offers time complexity of O(1). On the other hand, HR has been proven to give the best communication costs of the order of log(b) for b-bits communication. Therefore, *Sarve* is a step towards frequency oracles that exhibit how privacy provisions of existing methods can be combined with those of fake data to achieve statistical results comparable to the original data. *Sarve* also implemented an adaptive solution enhanced from the work of Arcolezi et al. The use of RAPPOR was found to provide better privacy-utility tradeoffs for specific privacy budgets in both high and general privacy regimes.

## Introduction

The data collected from users is an essential resource for the improvement of services provided by hosted platforms. The user data is aggregated to compute statistical properties that support or reject a hypothesis, drive analytics, and train Artificial Intelligence (AI) models (Lee and Clifton 2011; Tanwar et al. 2020a, 2021). This collection of user information is a double-edged sword since its misuse by malicious parties can infringe on the individual privacy of the participants. Data breaches that spanned from the leak of Netflix users’ information to recent incidents like the sale of login credentials of Zoom users or attacks on popular social platforms like Twitter, LinkedIn, and Whisper (Wagenseil 2020; Huang and Ban 2020; Memon et al. 2020; Boulanger, 2018) have resulted in a loss of trust among the users. The call for stricter laws resulted in regulations like General Data Protection Regulation (GDPR), Data Governance Act, ePrivacy Regulation, Do Not Track legislation, and so on (Härting et al. 2017; Tanwar et al. 2020b; Kirsch 2011; Mahanti 2021; Grafenstein et al. 2021; Xue et al. 2021). These regulations have thereby promoted user privacy needs from being an additional feature to a mainstream requirement from products and services.

The development of data privacy requirements has fueled research methods that ensure the release of user data with privacy guarantees. A privacy guarantee aims to protect against the disclosure of an individual's identity, or any of their attribute that might result in the identity disclosure (Ahamad et al. 2020). An attribute set *A* = {*A*_1_, *A*_2_, …,* A*_D_} belonging to an individual may contain information of varying sensitivities. For instance, the pregnancy status of the individual in a medical record may be more sensitive than their family history of Diabetes. An aggregator who collects such data would be aware of the patients’ identities but not their sensitive information. Therefore, privacy provisions need to be designed such that the presence of a specific individual cannot be discovered from a privatized record.

Privacy provision methods could be syntactical and simply suppress or generalize the identifying attributes and release the modified information. Some examples include k-anonymization, l-diversity, and t-closeness (Tu et al. 2018; Mehta and Rao 2019; Kacha et al. 2021). The information with suppressed fields could still be prone to inference attacks (Gatha et al. 2020a). Consequently, a semantic strategy was formulated to release user data in the form of query results. Differential privacy (Dwork et al. 2010a, b) is one such popular technique that provides individuals with plausible deniability of their participation in a data record.

Differential privacy (DP) allows for the addition of calibrated noise to ensure that the presence or absence of an individual in a record does not affect the query results. The most generic form of DP involves the addition of noise to the collected record of user information and subsequent queries are run on the modified data (Dwork et al. 2010a, b). But another of its variant named local differential privacy (LDP) (Xiong et al. 2020; Yang et al. 2020) has been accepted more widely. In this scheme, noise can be added at the end of the individuals who comprise the record. Noise addition at the source can prove to be particularly beneficial if the data aggregator is vulnerable to breaches or is a less trustworthy third party (Wang et al. 2018a, b; Wang et al. 2021a, b).

The estimation of statistical distribution from samples of a population is a classical problem (Nguyên et al. 2016; Xu et al. 2020) and one of the major metrics to determine the utility of privatized data. The attributes that belong to user data could be of different types that include but are not limited to textual, numeric, or temporal. In the case of numerical attributes, the expected statistical estimation property is governed by their type. For discrete or categorical attributes, statistical distribution estimation is the evaluation of the frequency of underlying discrete distribution (Zheng et al. 2021). For continuous numerical attributes, mean estimation is the most popular statistical task. The scope of this paper and the proposed solution is concerned with the frequency estimation of categorical attributes of locally-privatized data.

LDP has found wide acceptance and incorporation into mainstream solutions such as the collection of telemetry data by Windows10, collection of usage patterns by Google’s Chrome browser, Harmony by Samsung, and incorporation in Apple’s iOS among many (Kenthapadi et al. 2019). Moreover, research has enriched it further with the addition of robust techniques like randomization, shuffling, sampling, and k-anonymization (Zhao et al. 2019).

In comparison with the innovative approach of Federated learning, LDP has been found to give lower misclassification rates for a large population. It also offers the additional benefit of flexible control over privacy budgets (Zheng et al. 2020). Nonetheless, data privatized by LDP is prone to inference attacks especially if the query mechanism is hosted in an interactive setting. An adversary intent on executing a disclosure will have the necessary computation power and skills. With repeated and carefully crafted queries, they can generate a subset of results that can point to an individual's presence in the record (Rahimian et al. 2020). This problem is more relevant in the case of heavy hitters (Zhu et al. 2020), where the data for certain individuals is present more frequently in the distribution. Another consideration when DP is employed is the balance of privacy achieved by noise addition and the utility of the perturbed data (Li et al. 2021).

Synthetic data, also known as fake data, is fast gaining prominence as means of achieving privacy guarantees. Datasets that have similar statistical properties of distributions and correlations can be published in place of real or privatized real data (Campbell 2019). The advantages include higher privacy guarantees due to its immunity to inference attacks by reverse-engineering or background knowledge possessed by the adversary. But the technology is still in a nascent stage. There is scope to develop more efficient solutions that are not computation heavy or divulge real data that was used for its generation (Hittmeir et al. 2019; Emam et al. 2021).

In this paper, the combination of LDP through randomization and fake data has been enhanced to provide more robust privacy guarantees. The frequency estimation of the data privatized through the proposed framework was compared against the distribution of the original data for high-privacy as well as general-privacy regimes.

### Motivation and techniques overview

The major concern that drives frequency estimation of privatized data is the design of a mechanism *M* to achieve minimal variance with respect to the original data. Wang et al. (2018a) introduced the term Frequency Oracle (FO) to denote a pair of algorithms < τ, υ > where mechanism τ outputs the perturbed or randomized attributes and υ is the estimation method. The algorithm υ is used by the aggregator for statistical computation over privatized data. Research has categorized FO into four major types namely, direct perturbation such as the use of randomized response (Warner 1965; Kairouz et al. 2016a, b; Lin et al. 2018), hash-based methods like RAPPOR (Erlingsson et al. 2014), transformation-based methods such as Hadamard response (Acharya et al. 2018; Liu et al. 2020), and subset selection (Wang et al. 2016).

Existing research like Random Sampling plus Fake Data (RS + FD) solution by Arcolezi et al. (2021a, b) devised FOs that utilized methods like GRR and OUE. The variant of RAPPOR used in our experiment used bloom filters. Bloom filters are highly space-efficient data structures that offer time complexity of the order of O(1) (Erlingsson et al. 2014). HR, on the other hand, has been proven by Acharya et al. (2018) to have the lowest communication cost of the order of log(*b*) in a *b*-bits communication. RAPPOR and HR are therefore more suited than GRR to lower the computational and communication complexities of a smart system.

The framework proposed in this paper aimed to answer if the hash-based method RAPPOR and transformation-based method Hadamard Response could be used to construct efficient frequency oracles. A frequency oracle may function on a high or a generic privacy regime based on the privacy budget. Generic privacy regimes are commonly found in internet browsers and similar real-world applications. While the original RS + FD solution had only been tested on high-privacy regimes, we tested frequency oracles for general-privacy regimes as well. The incorporation of RAPPOR provided better privacy-utility tradeoffs for some instances of privacy regimes. In addition, HR was evaluated as a candidate mechanism τ and found to perform on par with GRR and OUE.

### Purpose and contribution

The RS + FD framework proposed by Arcolezi et al. utilized a combination of shuffling and sampling to achieve private frequency estimation for datasets containing categorical attributes. They also demonstrated how LDP combined with the use of fake data helped achieve a balanced privacy-utility tradeoff. We have summarized the workings of the approach in “Literature survey” section. The work by Arcolezi et al. used GRR and two variants of OUE (Wang et al. 2017). Their solution showed better performance than the conventional methods of splitting and sampling. The RS + FD also proposed an adaptive solution that dynamically selected GRR or OUE depending on which method offered lower variance. The performance of their framework was compared with existing solutions for high privacy regimens only.

Numerous researches have identified randomization techniques and their advantages based on performance in different scenarios. Acharya et al. (2018) compared the performance of RR, RAPPOR, subset selection, and Hadamard response (HR). The evaluation metrics for their comparison were communication cost and decoding time required for estimating underlying probability distributions. HR is based on a local hashing mechanism that is symmetric across the *N* users. Among the candidates, it was found to offer the best communication cost of *log b* + 2 bits per user, where *b* measured the size of the bit vector of the entity to be transmitted. Recent work by Chen et al. had also built on HR to propose a Recursive Hadamard Response (RHR) that facilitates privacy guarantees for the case of distributed learning (Chen et al. 2020).

In another paper, Acharya et al. (2019) have discussed how the construction of the Hadamard matrix incurs large memory costs. RAPPOR is a unary encoding-based method and is used as a primary LDP protocol by Google's Chrome browser. Le and Zia (2021) also carried out a comparative analysis and discovered that RAPPOR gave the best performance for benchmark datasets in high privacy regimes. This led us to consider the two mechanisms Hadamard Response and RAPPOR as randomization candidates τ to enhance the RS + FD solution by Arcolezi et al. We borrowed the fast implementation of RAPPOR by Cormode et al. (2021) and modified the HR implementation from Acharya et al. and tested their applicability to developing FOs extended from RS + FD. The details of the use of HR and RAPPOR can be found in next sections respectively.

The contributions of this paper can be summarized as:

- We introduce the use of RAPPOR in combination with fake data to facilitate frequency estimation of multidimensional datasets under high-privacy as well as generic privacy regimens.
- We introduce the use of Hadamard Response in combination with fake data to facilitate frequency estimation of multidimensional datasets under high-privacy as well as generic privacy regimens.
- We extended the RS + FD solution by Arcolezi et al. through the incorporation of RAPPOR to compare privacy-utility tradeoffs offered by the new candidates. The enhancement was tested for high-privacy as well as generic privacy regimens. Furthermore, the utility of privatized data was tested using real-world as well as synthetic datasets. The proposed framework that incorporated advantages of multiple techniques has been named *Sarve*, a Sanskrit word that means ‘all together’.

### Paper organization

The problem addressed in this paper and our contributions have been discussed in “Motivation and techniques overview” and “Purpose and contribution” sections, respectively. With the introduction to the premise of this paper, Sect. 2 recaps the preliminary information required by the reader, and “Literature survey” section contains the literature survey of the concerned research. “Proposed methodology” section explains the proposed methodology with the architecture of *Sarve* in “Overview of sarve” section. “Application of RAPPOR in a frequency oracle”, “Application of Hadamard response in a frequency oracle”, “Enhancement of adaptive RS+FD” sections detail the privacy-enhancing mechanisms RAPPOR and HR used in *Sarve*. In “Experimental results” section, we provide the implementation details of the solution. The metrics used for the evaluation are mentioned in “Evaluation metrics” and “Experimental setup” sections explains the experimental setup. The results achieved by the proposed solution and discussion are in “Results and discussion”. We have concluded the findings and scope for future work in “Conclusion” section.

## Preliminary

### Notations

Through the course of this paper, the constant $\upvarepsilon$ denotes a privacy budget. The significance of its values has been discussed in further sections. RS + FD is the name of the solution by Arcolezi et al. which formed the basis of our research. The data to be privatized was assumed to contain *N* observations each belonging to an individual. Each of these observations had categorical attributes that were allowed a set of allowed domain values *A* = {*A*_1_,* A*_2_, …,* A*_D_}. The data aggregator would aim to get the frequency of each value in set *A*.

### Local differential privacy

Local differential privacy has emerged as a well-suited technique for systems that aggregate sensitive user information. Its popularity can also be attributed to the fact that it can be achieved through a wide selection of privatizing mechanisms. These mechanisms can be selected for specific requirements like computational costs, data dimensionality, desired privacy regimes, and communication overheads. An $\upvarepsilon$-LDP is satisfied by a privatization mechanism *M* if it satisfies the probability condition,

1 $$\Pr [M(t) = \varphi ] \le e^{\varepsilon } \cdot \Pr [M(t^{\prime } ) = \varphi ]$$

where *t* and *t*′ are sets of values that differ by one element only and φ is the output after *M* has been applied to *t* and *t*′. If *t* and *t*′ are two records of user information that differ by the presence of an individual, then *M* will be applied to each entry in *t* and *t*′. The possible output of *M* identified as φ will differ by a factor of *e^*$\varepsilon$ for both the user records.

The privatization mechanisms include perturbation or randomization. Assuming *M* to be a randomization method, if the private information *t* is denoted as a set *T* with *k* possible values such that T = [*k*] =* {0, 1, …, k − 1}*, *M* will map *t Є T* to *d Є φ* with a probability *P*(*d|t*). The output value *d* is the privatized sample that is shared by an $\upvarepsilon$-LDP-protected system. The privatization probabilities can be again shown as a factor of *e^*$\varepsilon$ like:

2 $$\mathop {\sup }\limits_{d \in \varphi } \frac{P(d|t)}{{P(d|t^{\prime } )}} \le e^{\varepsilon }$$

The constant $\upvarepsilon$ is called a privacy budget. As specified by Eq. (2), smaller values of $\upvarepsilon$ put stringent restrictions on the mechanism *M* and therefore dictate a highly privatized output set. Conversely, bigger values of $\upvarepsilon$ result in low or general privacy regimes (Kairouz et al. 2016a; Ye et al. 2019).

LDP mechanisms are particularly advantageous since they are understandable by novice users. Additionally, no original information needs to be shared with the data aggregator which lowers the legal and technical costs of ensuring privacy at the aggregator's end (Le and Zia 2021). The methods employed by LDP systems are relatively simpler to execute and restrict the communication costs of transmitting multidimensional data (Wang et al. 2019a, b, c). Despite providing strict privacy guarantees and multiple advantages, LDP mechanisms are vulnerable to adversarial manipulation. As proven by existing research, LDP-protected systems with high privacy regimes hosted in non-interactive settings are also vulnerable to manipulation attacks (Cheu et al. 2019a, b). Thereby, opening avenues for its use in combination with other privacy-enhancing techniques such as synthetic data.

### Privacy amplification methods

The privacy guarantees provided by LDP can be further amplified by the use of methods like iteration, sampling, and shuffling. The privacy enhancement by iteration is based on how learning algorithms work. They create intermediate solutions and iteratively improve upon them using data points. It was proved that withholding the intermediate results produced by learning processes such as stochastic gradient descent can amplify the privacy guarantees (Feldman et al. 2018; Sordello et al. 2021).

Privacy amplification through sampling utilizes the existing technique of data sampling where partial contents are selected from the complete set (Balle et al. 2018). It is a resource-saving method that has been widely researched and is available in different variants. Li et al. presented that data sampling can minimize the disclosure potential of user information while being true to the data properties (Li et al. 2012). Feldman et al. showed that randomly shuffling the data records that are to be input to differentially private local randomizers improved the privacy guarantees of the system (Feldman et al. 2020; Erlingsson et al. 2019; Cheu et al. 2019a, b).

### Frequency estimation

Real-world data that needs privatization is generally multidimensional and thereby adds more concerns to otherwise simpler computations. For the scope of this research paper, we focused on the frequency estimation of categorical attributes of multidimensional datasets. Due to the higher number of attributes, concerns for privacy budget $\upvarepsilon$ also gain prominence (Wang et al. 2019a, b, c; Xu et al. 2020). The randomization mechanism *M* used to apply LDP to each user in the multidimensional dataset works in two ways. The first strategy divides the privacy budget $\upvarepsilon$ over all of the attributes and the user then shares all of the randomized attribute values with the aggregator. In the second approach, a single attribute is selected through random sampling, and $\upvarepsilon$ is solely applied to this attribute. Existing research has shown that while sampling and randomizing an attribute achieves a better privacy-utility trade-off, it might not be fair in case the sampled attribute is less sensitive than the others (Arcolezi et al. 2021b, a). Therefore, we explored the potential of improving the performance of LDP facilitated by the random sampling method.

The data released after privatization is used for statistical computation, and frequency estimation is one of the most common statistical goals for privatized categorical data. Assuming that the record consists of *N* users, where each user entry has only one value among the set of allowed domain values *A* = {*A*_1_,* A*_2_, …,* A*_D_}. The data aggregator would aim to get the frequency of each value in set *A*, denoted by.

3 $$f(A_{i} ) = \frac{{count(A_{i} )}}{N},\quad 1 \le i \le D$$

where *count(A*_*i*_*)* is the number of users who had reported attribute value of *A*_*i*_, and is computed over the output of privatization mechanism *M*.

The computation of statistical properties of the aggregated data holds potential for privacy leaks. In the context of this paper, we focused on privacy leaks due to frequency estimation. The aggregator who collects the privatized data belonging to individuals would estimate the count of persons for each of the values in domain A. This collecting and the aggregating party is aware of the individuals who comprise the dataset but not the values of their private data (Arcolezi et al. 2021b, a). For instance, a diagnostic clinic application may collect protected health information during registration that could include pregnancy or HIV status. Since pregnancy status is limited to a very small demography of females between the ages of 13–45, the reported frequency for this field will be noticeable compared to other attributes. The computed frequency for this field when combined with other attributes like age or choice of further tests can help an adversary uncover the individual’s identity.

The potential for privacy leaks can be further elucidated when we consider the use of frequency estimation to identify heavy hitters. An attribute is labeled as a top-f heavy hitter if its estimated frequency is among the top f frequencies among all of the calculated values (Wang et al. 2021b). The password preference of people is one such use case. Persons belonging to younger generations may prefer to use the name of their favorite band or sports team as a password. Or, it is common knowledge that people end up using simple passwords like ‘Password123’, ‘Password@123’, or ‘P@ssword123’. An adversary could shortlist the easiest password to crack from the aggregated passwords of a user group (Naor et al. 2019). This is a particularly worrisome situation since many IoT devices are shipped with default passwords that are not changed by the users. Such leaks of passwords hold the potential to cause widespread botnet attacks like the Mirai (Naor et al. 2019). Therefore, there is a pressing need to research privacy-aware privatization and aggregation methods and this paper is a step to address it.

## Literature survey

Differential privacy (DP) emerged as a frontrunner for semantic privacy definitions after syntactic privacy protection methods were found vulnerable to disclosures (Gatha et al. 2020b). Initial implementations of DP were based on a central model where the aggregator would add noise to the collected data and share it for further analysis (Dong et al. 2019). This required trust to be placed on a third-party and statistical computations could also cause identity disclosures (Kifer et al. 2020). With the adoption of DP into cloud-hosted services, the onus of privatization shifted to the users. This variant of DP called Local Differential Privacy provided individuals with more control over their privacy budgets (Wang et al. 2021a).

Over time, LDP has been adopted by many widely used platforms and service providers, thereby fueling research on its evolution. The evolution was aimed at addressing requirements such as multi-dimensionality and resulting complexities, communication costs, or decoding mechanisms (Acharya et al. 2018). The nature of the attributes present in the dataset to be privatized also governs the statistical computations. For instance, categorical attributes are computed for frequency estimation. These can be subsequently used to predict heavy hitters and balancing of user privacy with aggregator accuracy (Lopuhaä-Zwakenberg et al. 2020). For continuous numeric attributes, the statistical computations involve mean estimation. This particular class of attributes is a separate research subject (Xue et al. 2021; Wang et al. 2019a, b, c).

Akin to any technology that has been deployed to real-world applications, LDP also has shortcomings and vulnerabilities. It has been a known target of poisoning and other attacks due to the collection of data from multiple sources (Cao et al. 2019). This may allow an adversary to inject their compromised data and corrupt the collected records (Cheu et al. 2019a, b). Moreover, if hosted on an interactive platform, LDP privatized results are accessible for indefinite permutations of queries (Dwork et al. 2010a, b; Joseph et al. 2018). The biggest motivation for the adversary is the use of real user data that was randomized or perturbed by an algorithm. Reverse-engineering or statistical inferences are highly probable threats. A potential workaround to this problem has been proposed through the use of synthetic or fake data (Abay et al. 2018).

The generation of fake data is fast gaining popularity due to many reasons such as the need for precise labels for deep learning models (Alkhalifah et al. 2021; Hoffmann et al. 2019) or fears of identity disclosure by data holders (Snoke et al. 2018). The need for synthetic datasets became more prominent during the SARS-Cov-2 pandemic since the novel infection translated to a shortage of datasets to train medical AI models (Emam et al. 2021; Bautista and Inventado 2021).

This research domain is in the nascent stage. The construction of synthetic datasets and their utility metrics have become an exciting research problem (Snoke et al. 2018). Further exploration of this avenue also compared the protection provided by fake data against conventional methods like k-anonymization (Hittmeir et al. 2020). Recent findings showed that synthetic datasets having similar statistical properties as real data may offer privacy protection against inference attacks. The protection was on par with conventional anonymization methods (Stadler et al. 2022). Therefore, the generation of differentially-private synthetic data has been proposed as one of the solutions (Vietri et al. 2020; Quick 2021). While some methods have aimed at mitigation of bias in datasets (Ghalebikesabi et al. 2021), others have compared differentially-private synthetic data against baseline DP models for similar privacy budgets (Rosenblatt et al. 2020; Snoke and Slavković 2018).

Frequency estimation is a classic use case for privacy protection since analytics translate observations into the frequency of relevant attributes. Such statistical translation helps find relevant behavior such as the heavy hitters (Ben Basat et al. 2020; Pekar et al. 2021; Wang et al. 2021b; Zhao et al. 2022), frequent items (Luna et al. 2019; Wang et al. 2018a; Djenouri et al. 2018, 2019; Rouane et al. 2019; Li et al. 2019), or finding the marginals (Zhang et al. 2018; Cormode et al. 2018; Xue et al. 2021; Wang et al. 2019a, b, c). While individuals who comprise the records require plausible deniability from participation in the record, the statistical values should not deviate to extremes. Metrics and lower-bounds set for privacy-utility tradeoffs (Lopuhaä-Zwakenberg et al. 2019, 2020) are guiding lights for privacy provision methods. Numerous methods have therefore emerged that offer flexible privacy suited for different data release and trust regimes. Data can be released as marginal tables which may be in the form of count or range query answers (Wang et al. 2019a, b, c). Such privacy methods focus on the sensitivity and size of the dimensions and place zero trust in the aggregator. In contrast, some methods may assume the aggregator’s knowledge of the distribution followed by the attributes (Jia and Gong 2019). In a previous section, we have discussed the advantages offered by sampling to improve the offered privacy provisions. Privacy-preserved frequency estimation has also been achieved with a combination of sampling and Multi-Party Computation (MPC), a cryptographic protocol (Yang et al. 2021).

## Proposed methodology

### Overview of Sarve

**Fig. 1:**
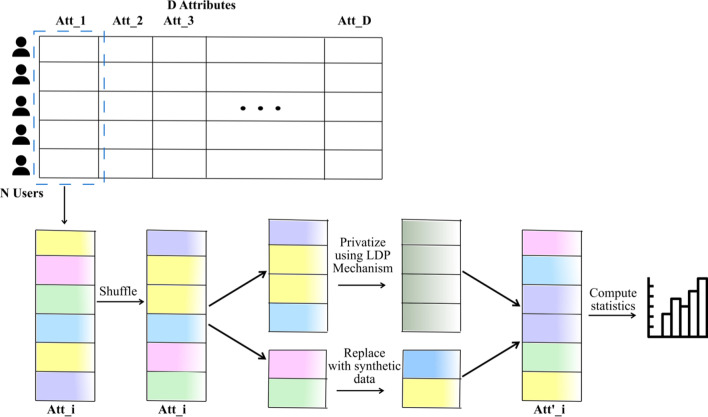
A schematic of the RS + FD framework proposed by Arcolezi et al. that formed the basis of the proposed solution *Sarve*

*Sarve* is an enhancement of the RS + FD framework through the incorporation of RAPPOR as randomization techniques. As part of the analysis, the application of Hadamard Response to frequency oracles was also tested. The RS + FD framework was conceptualized for an LDP system that comprised of *N* users who send their privatized data to an aggregator. Each user dataset had been assumed to contain a set of *D* categorical attributes identified by the set *A* = {*A*_1_, *A*_2_, …, *A*_D_}. In the case of RS + FD, the randomization mechanism *M* could either be GRR, OUE-R, OUE-Z, or the adaptive solution ADP. In *Sarve*, the randomization mechanism *M* has been extended to include RAPPOR and Hadamard Response. Additionally, RAPPOR had been added as a candidate to ADP. We have summarized the methodology employed by RS + FD and thus *Sarve* in Fig. 1. In the next section, the incorporation of the new randomization candidates has been discussed in detail.

As described in “Purpose and contribution” section, the framework utilized a combination of sampling and fake data to privatize user records. Additionally, the tuples were shuffled before being input into sampling algorithms. This method has been proven to amplify privacy. If the data record contained *N* entries for each user, each described by *D* attributes, the workings of the adopted methodology can be seen in Fig. 1. A list comprising of possible values for attribute *A*_*i*_ was first shuffled among the *N* users, then split into two parts. The first part was privatized through mechanisms that included GRR, OUE, RAPPOR, and Hadamard Response, in addition to the adaptive methods. The second set of the attribute values for the remaining users was replaced by fake data that had been randomly selected from the allowed domain values [0, *D − *1]. The privatized and replaced parts were merged to form the set of attribute values for statistical computation.

The RS + FD framework employed GRR and OUE techniques that are explained in the original paper. In the next section, we explain the incorporation of RAPPOR and HR and the resulting adaptive solution that has been named *Sarve*. The incorporation has been discussed in terms of the randomization and estimation algorithms of a frequency oracle.

### Application of RAPPOR in a frequency oracle

#### The randomization in frequency oracle using RAPPOR

We used the basic form of RAPPOR that has been proven to satisfy ε-LDP. It is based on unary-encoding and is suitable for highly-dimensional datasets. As explained in Algorithm 1, the attribute values that were to be privatized were first one-hot encoded to convert the input set *a Є [D]* to *R Є {0, 1}*^*D*^, where *D* is the domain size of the attribute value and *R*_*j*_ = 1 for *j* = *a* and *R*_*j*_ = 0 for *j ≠ a*. The bit vector *R* is privatized by independently flipping each bit of *R* with a probability *p* given by Eq. (4). The probability *q* of flipping *R*_*j*_ where *j ≠ a* is given by Eq. (5).

4 $$p = \frac{1}{{e^{\varepsilon /2} + 1}}$$

5 $$q = 1 - \frac{1}{{e^{\varepsilon /2} + 1}}$$

**Fig. 2:**
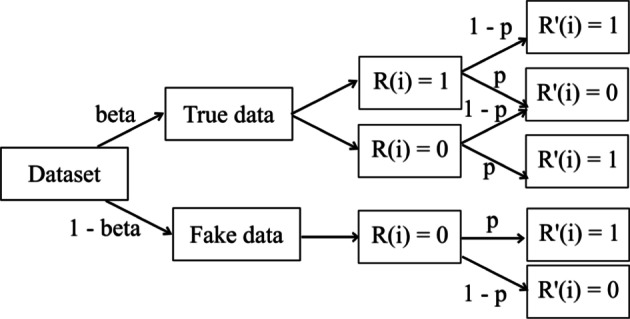
The probabilities associated with the use of RAPPOR as a privatization mechanism

Figure 2 illustrates the probabilities of sampling and flipping. Firstly, the part of attribute values to be privatized will be sampled with a probability *beta* = *1/D*. The flipping of the bits will be done with probability p.

The fake data comprised of a zeroes vector that was again randomized using RAPPOR. To contain the noise that could be added through the fake data, zeroes vector were used instead of randomly selected set of values. The implementation of this method was done per Algorithm 1.
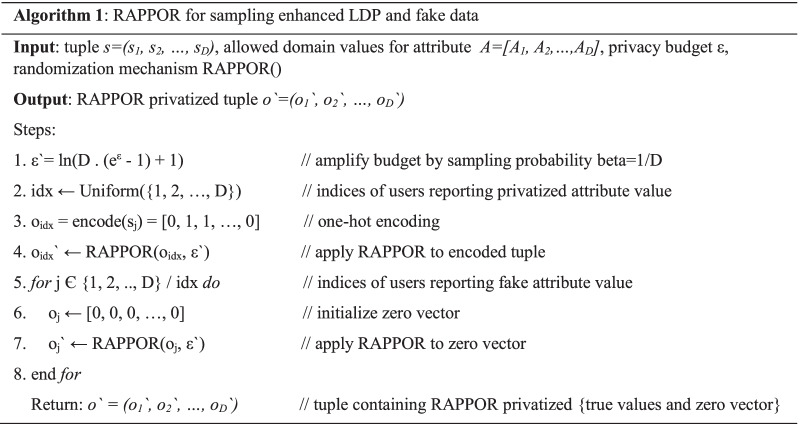


#### The estimation in frequency oracle

The frequency estimation method calculated the number of times the privatized data was reported. It is also a function of perturbation or randomization probability. For the randomizations carried out by probabilities p and q, Wang et al. (2017, 2018b) proposed the estimated frequency of privatized values as.

6 $$\hat{f}(A_{i} ) = \frac{{D \cdot (n_{priv} - N \cdot q)}}{N \cdot (p - q)}$$

where *D* is the domain size of the reported attribute *A*_*i*_, *n*_*priv*_ is the number of times the privatized attribute was reported, and *N* is the total number of observations in the record.

### Application of Hadamard response in a frequency oracle

*The randomization in frequency oracle using Hadamard Response*. The use of Hadamard Response for randomization is relatively recent among the other mechanisms mentioned in this paper. It is a hashing-based mechanism and therefore gives smaller decoding times as proven by Acharya et al. For randomization purposes, a Hadamard matrix is constructed in the form given by Eq. (7). For an attribute *A*_*i*_ that holds values within the allowed range [0, *D *− 1], the size of the Hadamard matrix is computed as *D* ≤ *D*′ ≤ 4*D. The Hadamard matrix *H*_*D*`_ = {1, − 1}^*D*^′^*XD*^′ will be constructed as.

7 $$H_{o} : = \left[ {\begin{array}{*{20}c} {H_{{o{/}2}} } & {H_{{o{/}2}} } \\ {H_{{o{/}2}} } & {H_{{o{/}2}} } \\ \end{array} } \right]\,\,\,with\,\,o = 2^{j} f\quad or\quad 1 \le j \le \log (D^{\prime } )$$

Additionally, it can be stated that *H*_*1*_ = [+ 1].

To privatize the attribute value *A*_*i*_ Є [0, *D *− 1], another value *Ai`*′ is selected from the domain size *D*′. This is done by choosing all of the elements from (*A*_*i* + 1_)th row index and the same block as *A*_*i*_ of the Hadamard matrix. The set of values returned from the Hadamard matrix can be called *S*_*A*_.

To privatize *A*_*i*_, an element from *S*_*A*_ will therefore be randomly selected with a probability.

8 $$p = \frac{{e^{\varepsilon } }}{{e^{\varepsilon } \cdot h + D^{\prime } - h}}$$

where *h* is the size of set *S*_*A*_. In the case of *Sarve*, we set *D*′ = *D*, *h* = 1, and *S*_*A*_ = *A*, therefore.

9 $$p = \frac{{e^{\varepsilon } }}{{e^{\varepsilon } + D - 1}}$$

**Fig. 3:**
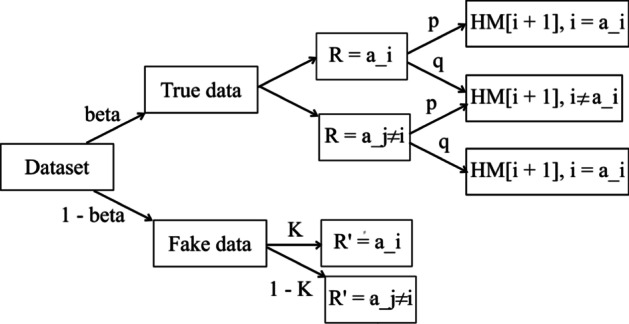
The probabilities associated with the use of Hadamard Response as a privatization mechanism

Thereby the solution was rendered similar to a randomized response mechanism. Algorithm 2 summarizes the steps that were implemented to realize the mechanism. Figure 3 shows the probabilities and resulting attribute values for the application of HR.
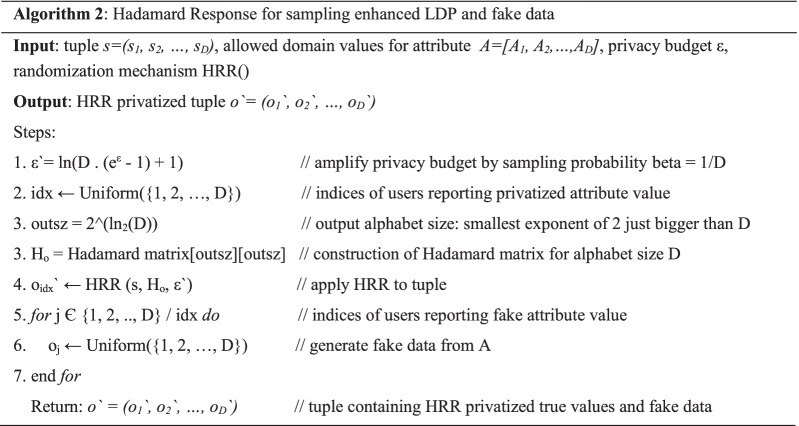

#### The estimation in frequency oracle

The frequency estimation strategy was the same as for the case of GRR since we had reduced the use of HR to a randomized response method. The probability *q* of not selecting the new symbol from *H*_*A*_ would be defined as.

10 $$q = \frac{(1 - p)}{{(D - 1)}}$$

The Eq. (6) would be modified to include the probabilities associated with the construction of set *H*_*A*_, and thus be used as

11 $$\hat{f}(A_{i} ) = \frac{{n_{priv} \cdot D \cdot A_{i} - N \cdot (D - 1 + q \cdot A_{i} )}}{{N \cdot A_{i} \cdot (p - q)}}$$

where *n*_*priv*_ is the numbers of times attribute value *A*_*i*_ had been reported and *N* is the total number of individuals present in the record.

### Enhancement of adaptive RS + FD

The user data in real-world conditions is multidimensional and uncertain. The variance is one of the most commonly used indicators to depict the utility of the privatized data. Therefore, an LDP protocol that results in lower variance can be dynamically selected among several candidates (Wang et al. 2017). Additionally, the mean square error (MSE) is a common evaluation metric for performance, and for estimators that are not biased variance can be measured as MSE (Wang et al. 2019b). In RS + FD, the authors dynamically selected between two candidates GRR and OUE to facilitate an adaptive LDP protocol. The results of experiments in this paper showed that the performance of Hadamard Response followed a trend similar to GRR but with bigger MSE values. Interestingly, RAPPOR performed better than GRR and OUE specifically for the general privacy regime. In *Sarve*, RAPPOR has been added as another candidate to further enhance the performance of the adaptive LDP protocol selection.

**Fig. 4:**
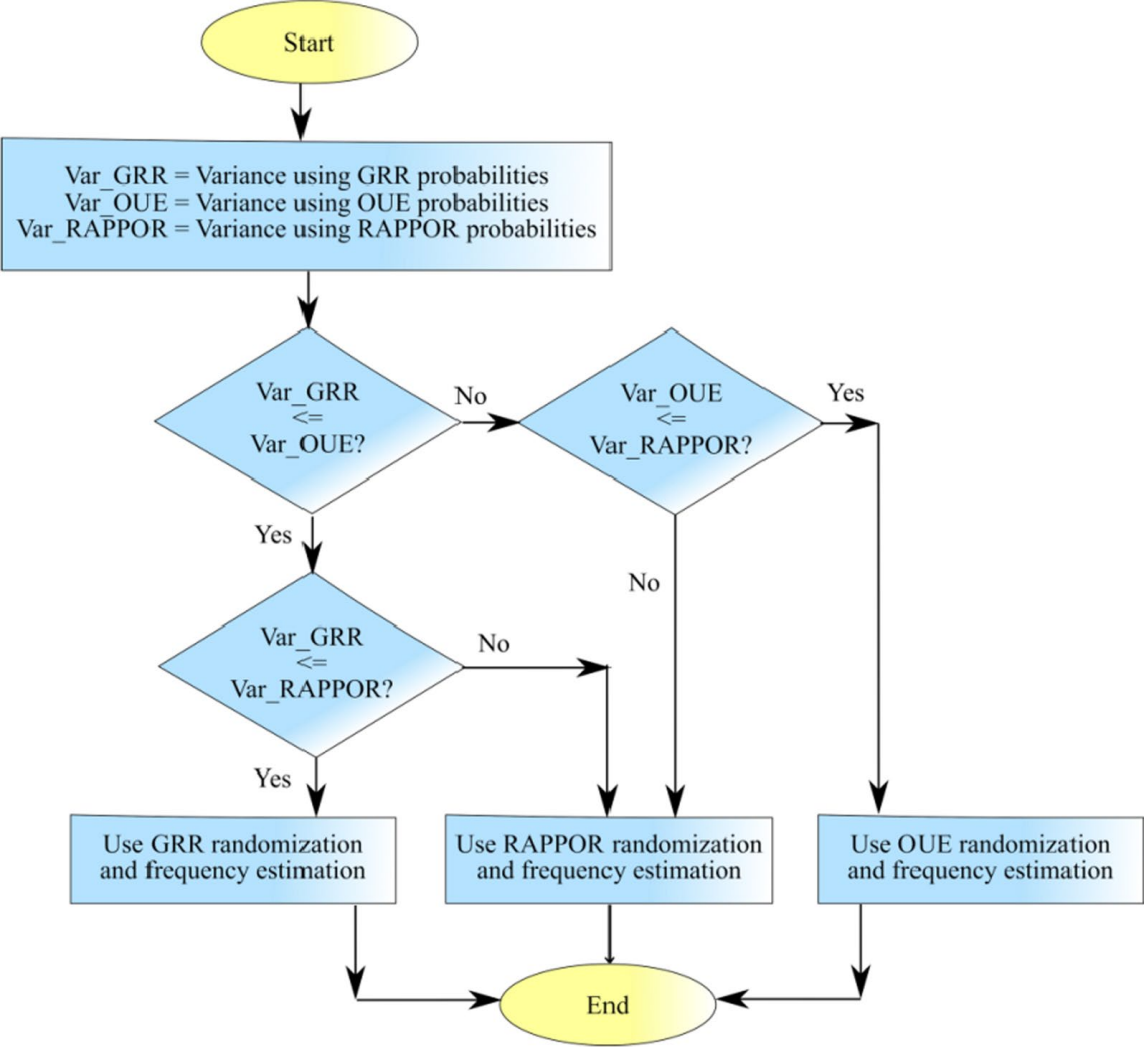
The flow of logic for an improvised adaptive approach to dynamic randomization using variance as the evaluation metric

As seen in Fig. 4, the algorithm looks for the randomization method that offers the least MSE, or in this case variance. The variance for the GRR method is calculated as.

12 $$Var(\hat{f}(A_{i} )) = \frac{{D^{2} \cdot \delta \cdot (1 - \delta )}}{{N \cdot (p - q)^{2} }}\quad where\quad \delta = \frac{1}{d} \cdot \left( {q + f(A_{i} ) \cdot (p - q) + \frac{(D - 1)}{{A_{i} }}} \right)$$

Since OUE and RAPPOR are both unary coding methods, the variance was formulated as.

13 $$Var(\hat{f}(A_{i} )) = \frac{{D^{2} \cdot \delta \cdot (1 - \delta )}}{{N \cdot (p - q)^{2} }}\quad where\quad \delta = \frac{1}{D} \cdot \left( {D \cdot q + f(A_{i} ) \cdot (p - q)} \right)$$

The algorithm computed the variances, selected the randomization method that gave lowest variance, and carried out privatization using the chosen randomization scheme.

## Experimental results

### Evaluation metrics

The statistical computations of this paper were focused on frequency estimation of categorical attributes belonging to multidimensional datasets. The estimated frequency of the privatized attribute values was compared against the original frequency distributions. The metric for the comparison was the mean squared error (MSE) as shown in Eq. (14).

14 $$MSE_{AVG} = \frac{1}{D} \cdot \sum\limits_{{j \in \left\lfloor {1,D} \right\rfloor }} {\frac{1}{{|d_{j} |}}(f(a_{i} ) - \hat{f}(a_{i} ))^{2} }$$

where *f(a*_*i*_*)* was the real frequency of the attribute *A*_*i*_ and was the estimated frequency of the attribute post-privatization.

Since randomization techniques had been employed for privatization, the algorithms were run 100 times, and mean MSE was computed for six different values of ε. Additionally, the algorithms were tested for regimes that mandated strong and general privacy. For the former case of strong privacy, values of ε were restricted to under 2, specifically a set of values were taken from RS + FD experiments as ε = [ln(2), ln(3), ln(4), ln(5), ln(6), ln(7)] = [0.6931, 1.0986, 1.3863, 1.6094, 1.7918, 1.9459]. The conditions for general privacy were defined by us with bigger values of ε = [2, 3, 4, 5, 6, 7].

### Experimental setup

All of the executions for different experimental setups were carried out on the operating system Linux Mint version 20 (Ulyana). The scripts were written in Python version 3.8.10 language and run in the Jupyter Lab development environment. The enhancements implemented as part of *Sarve* were tested on similar conditions as Arcolezi et al.’s RS + FD. The experimental setups had used real-world as well as synthetic datasets with different values of ε, number of observations, and number of categorical attributes, and allowed values for each attribute of the relevant dataset. The three real-world test datasets were the UCI adult income (Kohavi 1996), UCI nursery admissions (Olave and Rajkovic 1989), and MS-FIMU (Arcolezi et al. 2021a). The different combinations of test setups used for benchmarking have been summarized in Tables 1 and 2.

**Table 1:** The various parameter values that comprised the experimental setup were tested on real-world datasets

Experimental setup identifier	Values of ε	Dataset name	Number of observations = N	Number of categorical attributes = D	Number of allowed values for each attribute = A
ES_Real_1	[ln(2), ln(3), ln(4), ln(5), ln(6), ln(7)]	UCI Adult	45,222	9	[7, 16, 7, 14, 6, 5, 2, 41, 2]
ES_Real_2	[ln(2), ln(3), ln(4), ln(5), ln(6), ln(7)]	UCI Nursery	12,960	9	[3, 5, 4, 4, 3, 2, 3, 3, 5]
ES_Real_3	[ln(2), ln(3), ln(4), ln(5), ln(6), ln(7)]	MS-FIMU	88,935	6	[3, 3, 8, 12, 37, 11]
ES_Real_4	[2, 3, 4, 5, 6, 7]	UCI Adult	45,222	9	[7, 16, 7, 14, 6, 5, 2, 41, 2]
ES_Real_5	[2, 3, 4, 5, 6, 7]	UCI Nursery	12,960	9	[3, 5, 4, 4, 3, 2, 3, 3, 5]
ES_Real_6	[2, 3, 4, 5, 6, 7]	MS-FIMU	88,935	6	[3, 3, 8, 12, 37, 11]

**Table 2:** The various parameter values that comprised experimental setup tested on synthetic datasets

Experimental setup identifier	Values of ε	Dataset name	Number of observations = N	Number of categorical attributes = D	Number of allowed values for each attribute = A
ES_Syn_1	[ln(2), ln(3), ln(4), ln(5), ln(6), ln(7)]	50K_5D	50,000	5	[10, 10, 10, 10, 10]
ES_Syn_2	[ln(2), ln(3), ln(4), ln(5), ln(6), ln(7)]	50K_10D	50,000	10	[10, 10, 10, 10, 10, 10, 10, 10, 10, 10]
ES_Syn_3	[ln(2), ln(3), ln(4), ln(5), ln(6), ln(7)]	500K_5D	500,000	5	[10, 10, 10, 10, 10]
ES_Syn_4	[ln(2), ln(3), ln(4), ln(5), ln(6), ln(7)]	500K_10D	500,000	10	[10, 10, 10, 10, 10, 10, 10, 10, 10, 10]
ES_Syn_5	[ln(2), ln(3), ln(4), ln(5), ln(6), ln(7)]	500K_10D_NU	500,000	10	[10, 20, 30, 40, 50, 60, 70, 80, 90, 100]
ES_Syn_6	[ln(2), ln(3), ln(4), ln(5), ln(6), ln(7)]	500K_20D_NU	500,000	20	[10, 10, 20, 20, 30, 30, 40, 40, 50, 50, 60, 60, 70, 70, 80, 80, 90, 90, 100, 100]
ES_Syn_7	[2, 3, 4, 5, 6, 7]	50K_5 D	50,000	5	[10, 10, 10, 10, 10]
ES_Syn_8 ara>	[2, 3, 4, 5, 6, 7]	50K_10 D	50,000	10	[10, 10, 10, 10, 10, 10, 10, 10, 10, 10]
ES_Syn_9	[2, 3, 4, 5, 6, 7]	500K_5D	500,000	5	[10, 10, 10, 10, 10]
ES_Syn_10	[2, 3, 4, 5, 6, 7]	500K_10D	500,000	10	[10, 10, 10, 10, 10, 10, 10, 10, 10, 10]
ES_Syn_11	[2, 3, 4, 5, 6, 7]	500K_10D_NU	500,000	10	[10, 20, 30, 40, 50, 60, 70, 80, 90, 100]
ES_Syn_12	[2, 3, 4, 5, 6, 7]	500K_20D_NU	500,000	20	[10, 10, 20, 20, 30, 30, 40, 40, 50, 50, 60, 60, 70, 70, 80, 80, 90, 90, 100, 100]

The synthetic datasets were also constructed using Python scripts. As seen in Table 2, the generated distributions were uniform, except for non-uniform distributions labeled ES_Syn_5, ES_Syn_6, ES_Syn_11, and ES_Syn_12.

## Results and discussion

The performance of RAPPOR, Hadamard Response, and RAPPOR in the RS + FD adaptive solution called *Sarve* were compared with three main methods of RS + FD. The three RS + FD methods used for benchmarking the proposed solutions include:

- Spl[ADP] method had been implemented by randomly sampling a single attribute and spending the privacy budget ε on it. The adaptive approach selected between GRR and OUE for randomization was based on calculated variance.
- Smp[ADP] method was implemented by splitting the privacy budget ε across all categorical attributes. The adaptive approach selected between GRR and OUE for randomization was based on calculated variance.
- RS + FD[ADP] method that randomly sampled attributes and replaced some values with fake data. The adaptive approach privatized the true values of the attribute by choosing between GRR and OUE based on calculated variance.

The benchmarking aimed to show the performance of RAPPOR, HR, and *Sarve* such that the offered privacy guarantees and the utility of the privatized datasets were comparable to the above three methods. The results have been categorized based on testing through real-world and synthetic datasets.

### Results on real-world datasets

**Fig. 5:**
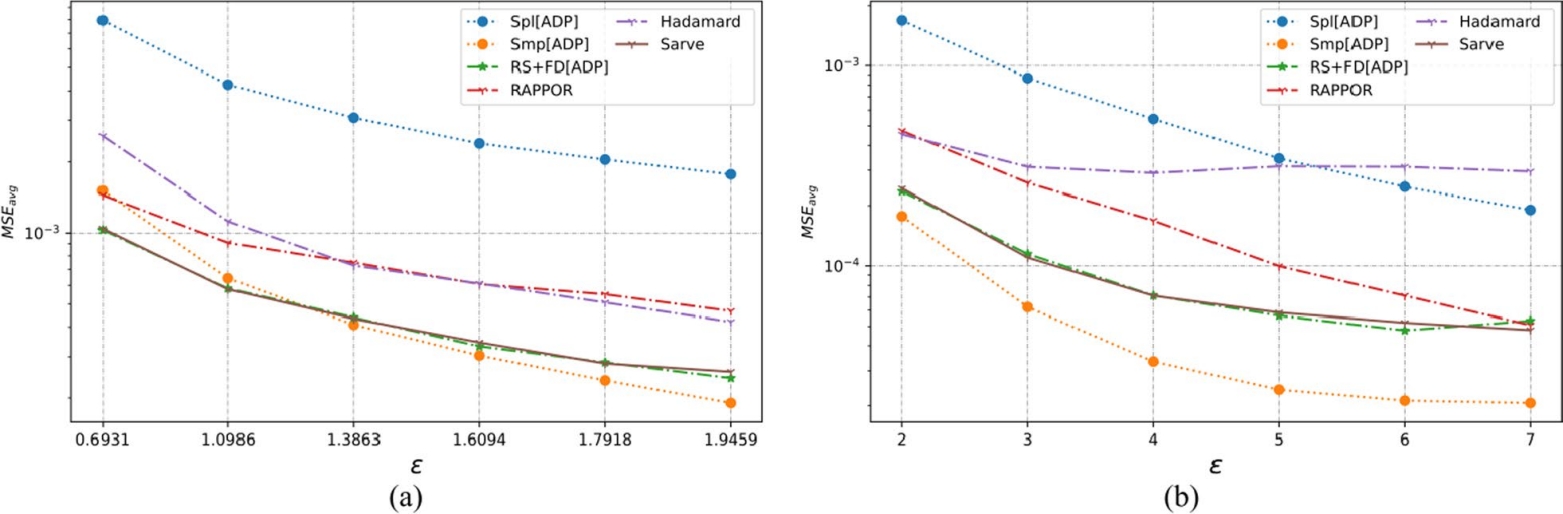
The MSE averaged over 100 runs for the UCI adult dataset privatized using different randomization techniques and fake data under **a** high privacy regime, experimental setup labeled ES_Real_1 in Table 1; **b** general privacy regime, experimental setup labeled ES_Real_4 in Table 1

**Fig. 6:**
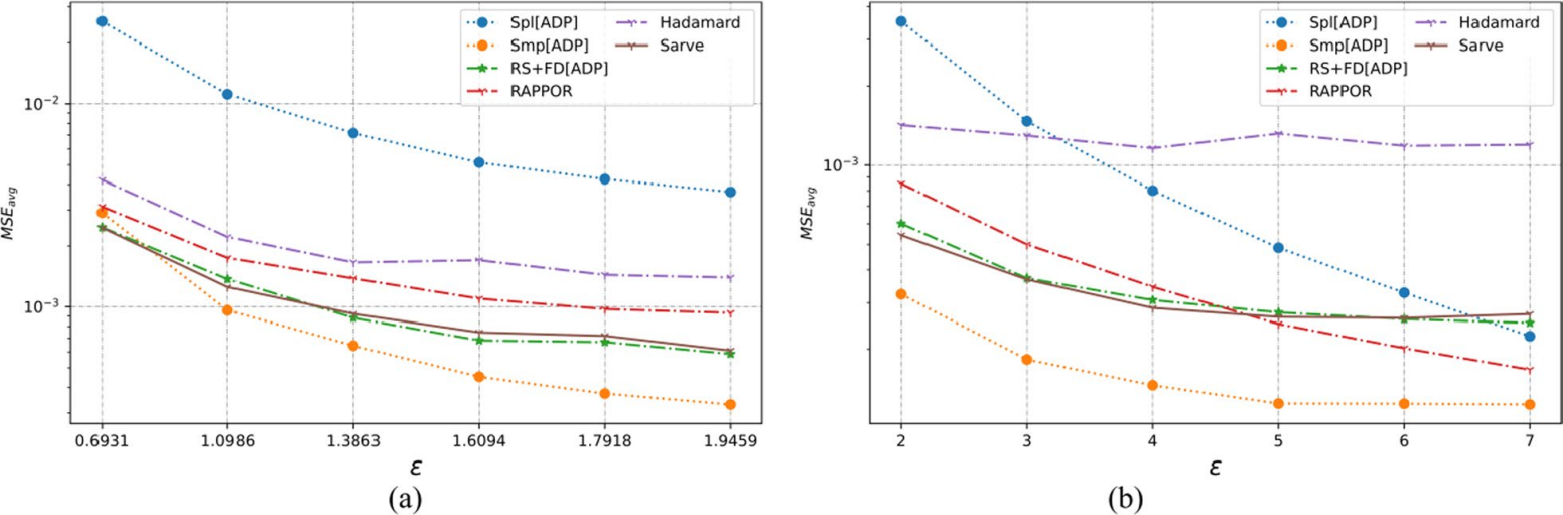
The MSE averaged over 100 runs for the UCI nursery dataset privatized using different randomization techniques and fake data under **a** high privacy regime, experimental setup labeled ES_Real_2 in Table 1; **b** general privacy regime, experimental setup labeled ES_Real_5 in Table 1

**Fig. 7:**
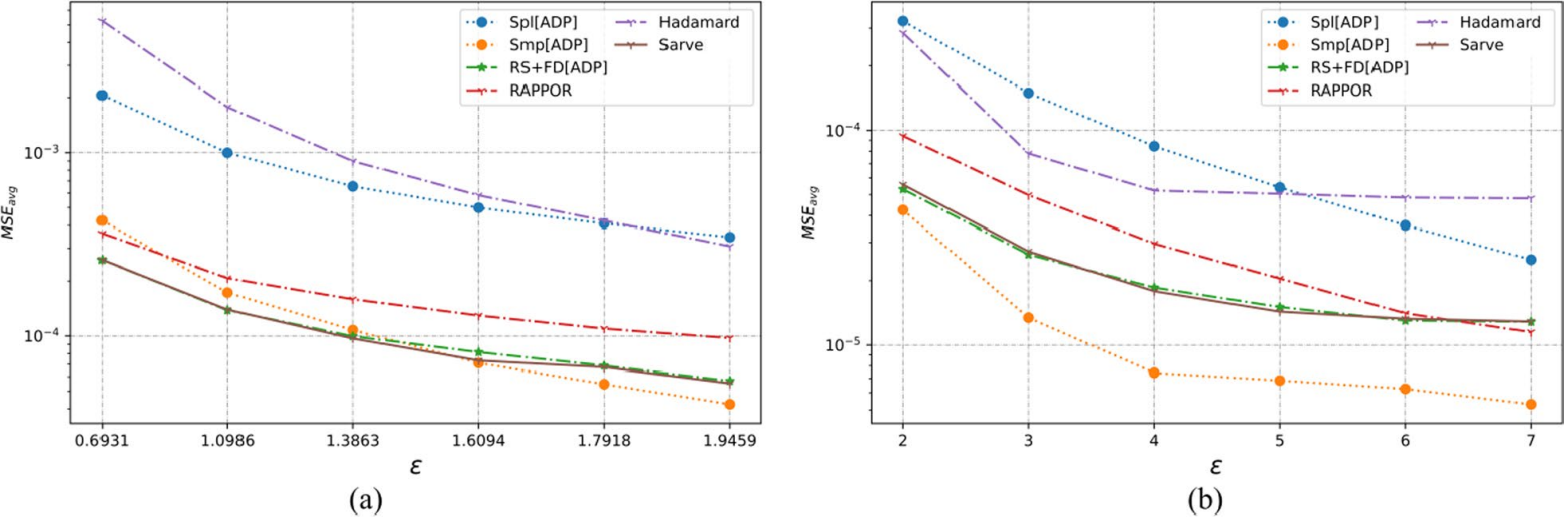
The MSE averaged over 100 runs for the MS-FIMU dataset privatized using different randomization techniques and fake data under **a** high privacy regime, experimental setup labeled ES_Real_3 in Table 1; **b** general privacy regime, experimental setup labeled ES_Real_6 in Table 1

The three multi-dimensional real-world datasets had a large number of observations with various types of domain values. As summarized in Table 1, the number of individuals varied from 12,000 to 88,000 with each dataset possessing six or more categorical attributes. The UCI Adult and MS-FIMU datasets had attributes that could take a value from a large set of values, i.e., set *A* was of the order of 10 or higher. The MSE averaged over a hundred runs for UCI Adult, UCI Nursery, and MS-FIMU datasets post-privatization by Spl[ADP], Smp[ADP], RS + FD[ADP], RAPPOR, Hadamard Response, and *Sarve* have been shown in Figs. 5, 6, and 7 respectively.

The effects of different factors like the number of observations attribute counts and domain values had affected the performance of randomization algorithms.

#### For high privacy regime

The UCI Adult dataset had 45 K observations and nine attributes. While Randomization through Hadamard response approximated the behavior displayed by GRR, privatization by RAPPOR was found to give lower MSE and thus a better utility. The use of RAPPOR in Arcolezi et al.’s RS + FD[ADP] did not degrade the performance of the algorithm.

The MS-FIMU dataset had the maximum number of observations at around 88 K and six attributes. The privatization offered by HR was not good but RAPPOR performed with a higher utility than the Smp[ADP] solution. *Sarve* created by the addition of RAPPOR to the RS + FD[ADP] enhanced the algorithm for the case when ε = 1.609.

The UCI Nursery dataset was the smallest among the real-world test data with the number of observations being 45 K and having nine attributes. While HR and RAPPOR alone performed better than Smp[ADP] but gave higher MSE than Spl[ADP]. The MSE values for *Sarve* and RS + FD were found to be lower than Spl[ADP] for ε = 0.693. Moreover, the use of RAPPOR enhanced the solution’s performance for ε = 1.09.

Overall, it can be said that RAPPOR performed better than benchmark solution Smp[ADP] but not Spl[ADP]. HR did not perform as well as RAPPOR in all of the test cases. Lastly, the addition of RAPPOR to RS + FD which was called *Sarve* was found to perform on par and better for certain values of ε.

#### For general privacy regime

For the datasets that had a large number of observations, that is the UCI Adult and MS-FIMU datasets, HR was found to perform better than Smp[ADP] for ε < 5. The performance of the method degraded as we moved to lower privacy regimes. The MSE achieved by RAPPOR was consistently better than HR and lower than those of RS + FD and *Sarve* for low privacy regimes. For the UCI Nursery dataset characterized by a smaller number of observations and high dimensionality of nine, HR did not perform well but RAPPOR gave low MSE for low privacy regimes. Overall, the privatization performance of *Sarve* was comparable to RS + FD[Adp] and better for specific values of ε.

The graphs were plotted for the MSE averaged over a hundred runs. For a clearer benchmarking between existing solution and the proposed work, the lowest MSE reported by the methods have been summarized in Tables 3, 4, and 5.

**Table 3:** The lowest MSE for the existing method and the enhancements which were tested on the UCI adult dataset

Method	ε = ln(2)	ε = ln(3)	ε = ln(4)	ε = ln(5)	ε = ln(6)	ε = ln(7)
RS + FD[ADP]	0.000596388	0.000325887	0.000278437	0.000183621	0.000162579	0.000126356
RAPPOR	0.000821897	0.000474918	0.000429617	0.000391613	0.000293406	0.000274562
Hadamard response	0.001347787	0.000483257	0.000394622	0.00029166	0.00023901	0.000161183
Sarve	**0.000559558**	**0.000315456**	**0.000243588**	0.000190493	**0.000150871**	0.00014343

Method	ε = 2	ε = 3	ε = 4	ε = 5	ε = 6	ε = 7
RS + FD[ADP]	0.000132441	0.000124503	3.01E−05	2.16E−05	1.39E−05	1.97E−05
RAPPOR	0.0002658	0.00013092	7.36E−05	5.38E−05	4.08E−05	2.55E−05
Hadamard response	0.000198588	0.000135697	0.000103984	9.55E−05	0.000137056	8.82E−05
*Sarve*	**0.000111824**	**5.53E**−**05**	3.16E−05	2.19E−05	1.54E−05	**1.60E**−**05**

The values in bold indicate privacy conditions when Sarve performed better than adaptive RS+FD and resulted in lower MSE between real and post-privatization estimated frequencies

**Table 4:** The lowest MSE for the existing method and the enhancements which were tested on UCI nursery dataset

Method	ε = ln(2)	ε = ln(3)	ε = ln(4)	ε = ln(5)	ε = ln(6)	ε = ln(7)
RS + FD[ADP]	**0.000734962**	0.000491035	**0.000220841**	**0.000250292**	**0.00030747**	**0.000189085**
RAPPOR	0.000981	0.000799	0.000441	0.000529	0.000483	0.000338
Hadamard response	0.00163	0.00075	0.000433	0.000659	0.000556	0.000396
*Sarve*	0.00087	**0.000445**	0.000417	0.000259	0.000387	0.000285

Method	ε = 2	ε = 3	ε = 4	ε = 5	ε = 6	ε = 7
RS + FD[ADP]	**0.000278**	0.000152	**0.000111**	9.31E−05	**8.91E**−**05**	**0.000109**
RAPPOR	0.000255	0.000219	0.000132	0.000112	8.07E−05	4.76E−05
Hadamard response	0.00048	0.000492	0.000452	0.000376	0.000378	0.000445
*Sarve*	0.000286	**0.000124**	0.000112	**9.06E**−**05**	0.000102	0.000119

The values in bold indicate privacy conditions when Sarve performed better than adaptive RS+FD and resulted in lower MSE between real and post-privatization estimated frequencies

**Table 5:** The lowest MSE for the existing method and the enhancements which were tested on MS-FIMU dataset

Method	ε = ln(2)	ε = ln(3)	ε = ln(4)	ε = ln(5)	ε = ln(6)	ε = ln(7)
RS + FD[ADP]	0.000108	**5.59E**−**05**	**4.64E**−**05**	**4.40E**−**05**	3.37E−05	2.97E−05
RAPPOR	0.000207	8.86E−05	8.01E−05	5.29E−05	5.78E−05	4.75E−05
Hadamard response	0.004249	0.001295	0.000635	0.00037	0.000263	0.000187
*Sarve*	**0.000105**	6.73E−05	4.78E−05	4.41E−05	**3.20E**−**05**	**2.72E**−**05**

Method	ε = 2	ε = 3	ε = 4	ε = 5	ε = 6	ε = 7
RS + FD[ADP]	**2.02E**−**05**	1.12E−05	**5.87E**−**06**	**3.90E**−**06**	**4.35E**−**06**	**3.10E**−**06**
RAPPOR	4.87E−05	2.28E−05	1.23E−05	8.58E−06	5.73E−06	4.91E−06
Hadamard response	0.000164	3.79E−05	1.88E−05	1.13E−05	1.72E−05	1.78E−05
*Sarve*	2.50E−05	**9.78E**−**06**	6.81E−06	4.64E−06	4.66E−06	3.21E−06

The values in bold indicate privacy conditions when Sarve performed better than adaptive RS+FD and resulted in lower MSE between real and post-privatization estimated frequencies

**Table 6:** The lowest MSE for the existing method and the enhancements which were tested on a synthetic 10-dimensional dataset having 50,000 records

Method	ε = ln(2)	ε = ln(3)	ε = ln(4)	ε = ln(5)	ε = ln(6)	ε = ln(7)
RS + FD[ADP]	0.000496	0.**000255**	**0.000198**	0.00015	**0.000114**	0.000111
RAPPOR	0.000807	0.00048	0.000325	0.00026	0.000247	0.000226
Hadamard response	0.000943	0.000312	0.000268	0.000215	0.000187	0.000151
*Sarve*	**0.000481**	0.000261	0.000207	**0.000145**	0.000118	**0.000106**

Method	ε = 2	ε = 3	ε = 4	ε = 5	ε = 6	ε = 7
RS + FD[ADP]	**9.65E**−**05**	4.51E−05	3.38E−05	**2.45E**−**05**	2.43E−05	**2.35E**−**05**
RAPPOR	0.000205	0.000112	6.63E−05	4.86E−05	3.06E−05	2.43E−05
Hadamard response	0.000177	0.000111	0.000126	0.000118	0.000102	9.77E−05
*Sarve*	0.000101	**4.39E**−**05**	**3.14E**−**05**	2.68E−05	**2.41E**−**05**	2.26E−05

The values in bold indicate privacy conditions when Sarve performed better than adaptive RS+FD and resulted in lower MSE between real and post-privatization estimated frequencies

**Table 7:** The lowest MSE for the existing method and the enhancements, tested on synthetic 20-dimensional dataset having 50,000 records

Method	ε = ln(2)	ε = ln(3)	ε = ln(4)	ε = ln(5)	ε = ln(6)	ε = ln(7)
RS + FD[ADP]	**0.000105**	**6.24E**−**05**	**4.30E**−**05**	**3.35E**−**05**	2.77E−05	**2.41E**−**05**
RAPPOR	0.000155	0.000121	9.77E−05	8.32E−05	7.35E−05	6.61E−05
Hadamard response	0.000159	7.82E−05	5.37E−05	4.32E−05	3.57E−05	3.27E−05
*Sarve*	0.000113	6.46E−05	4.51E−05	3.39E−05	2.77E−05	2.50E−05

Method	ε = 2	ε = 3	ε = 4	ε = 5	ε = 6	ε = 7
RS + FD[ADP]	2.35E−05	8.77E−06	4.36E−06	2.69E−06	2.17E−06	1.78E−06
RAPPOR	6.64E−05	3.93E−05	2.24E−05	1.31E−05	8.06E−06	5.27E−06
Hadamard response	3.29E−05	2.12E−05	1.90E−05	1.77E−05	1.58E−05	1.75E−05
*Sarve*	**2.27E**−**05**	**8.76E**−**06**	**4.33E**−**06**	**2.55E**−**06**	**1.96E**−**06**	**1.72E**−**06**

The values in bold indicate privacy conditions when Sarve performed better than adaptive RS+FD and resulted in lower MSE between real and post-privatization estimated frequencies

The attribute values followed non-uniform distribution

### Results on synthetic datasets

**Fig. 8:**
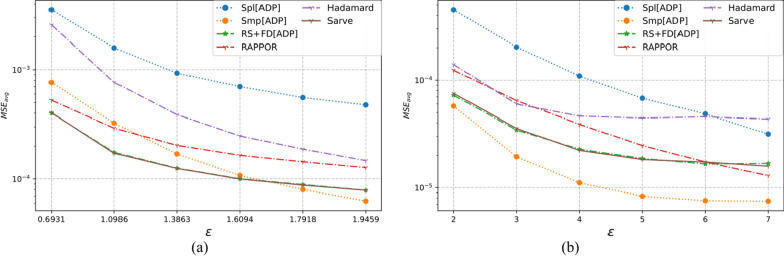
The MSE averaged over 100 runs for synthetic data with N = 50,000, D = 5 privatized using different randomization techniques and fake data under **a** high privacy regime, experimental setup labeled ES_Syn_1 in Table 2; **b** general privacy regime, experimental setup labeled ES_Syn_7 in Table 2

**Fig. 9:**
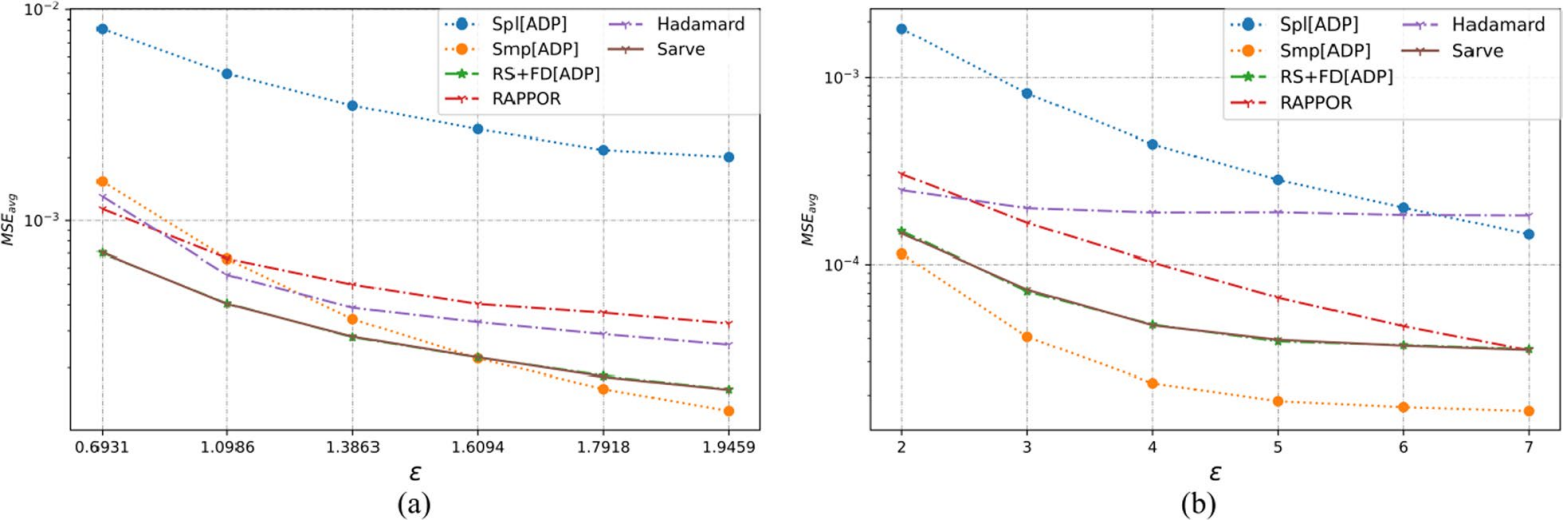
The MSE averaged over 100 runs for synthetic data with N = 50,000, D = 10 privatized using different randomization techniques and fake data under **a** high privacy regime, experimental setup labeled ES_Syn_2 in Table 2; **b** general privacy regime, experimental setup labeled ES_Syn_8 in Table 2

**Fig. 10:**
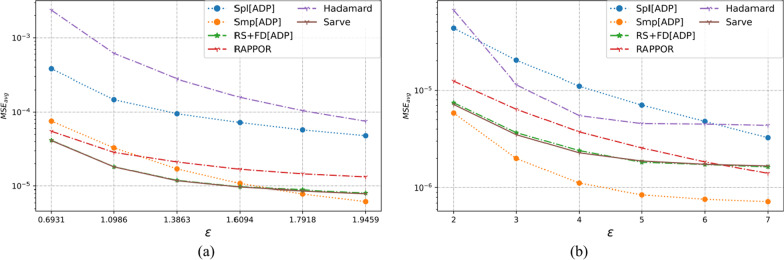
The MSE averaged over 100 runs for synthetic data with N = 500,000, D = 5 privatized using different randomization techniques and fake data under **a** high privacy regime, experimental setup labeled ES_Syn_3 in Table 2; **b** general privacy regime, experimental setup labeled ES_Syn_9 in Table 2

**Fig. 11:**
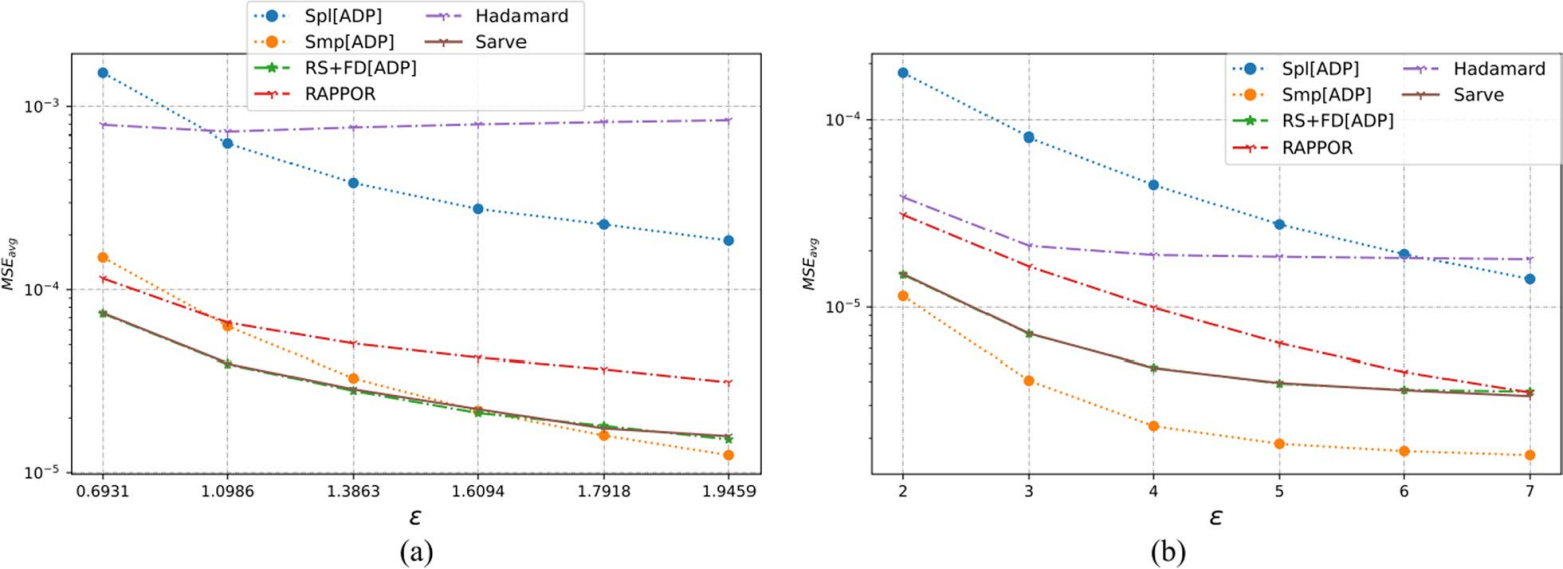
The MSE averaged over 100 runs for synthetic data with N = 500,000, D = 10 privatized using different randomization techniques and fake data under **a** high privacy regime, experimental setup labeled ES_Syn_4 in Table 2; **b** general privacy regime, experimental setup labeled ES_Syn_10 in Table 2

**Fig. 12:**
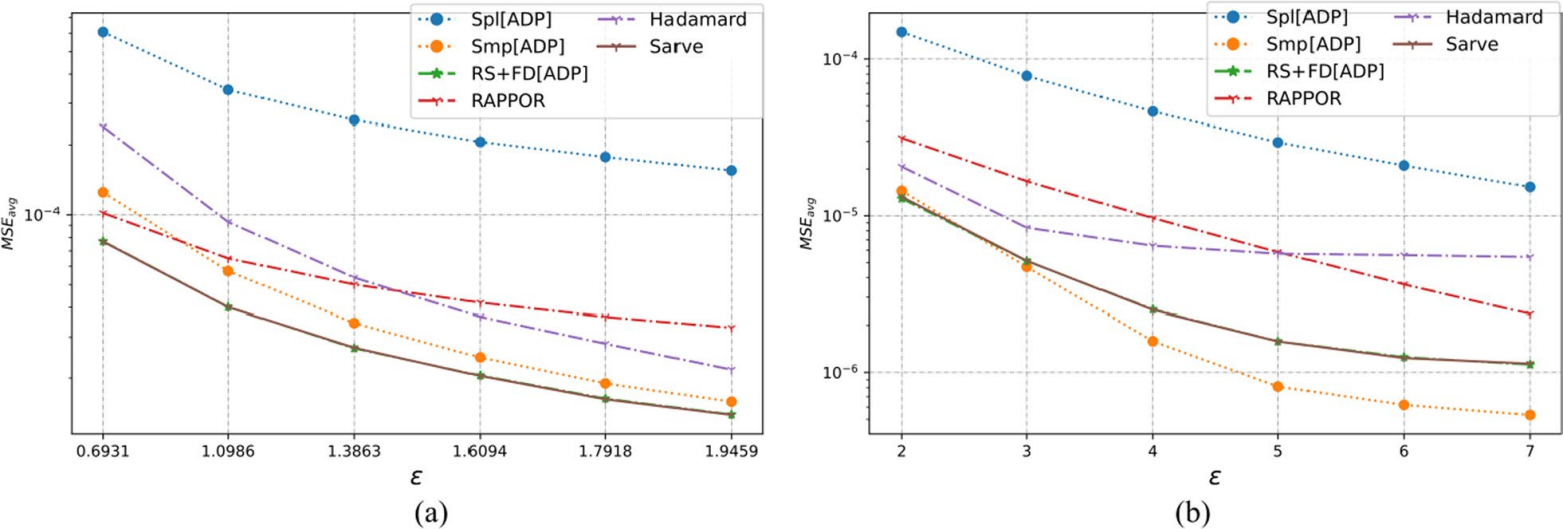
The MSE averaged over 100 runs for synthetic non-uniform data with N = 500,000, D = 10 privatized using different randomization techniques and fake data under **a** high privacy regime, experimental setup labeled ES_Syn_5 in Table 2; **b** general privacy regime, experimental setup labeled ES_Syn_11 in Table 2

**Fig. 13:**
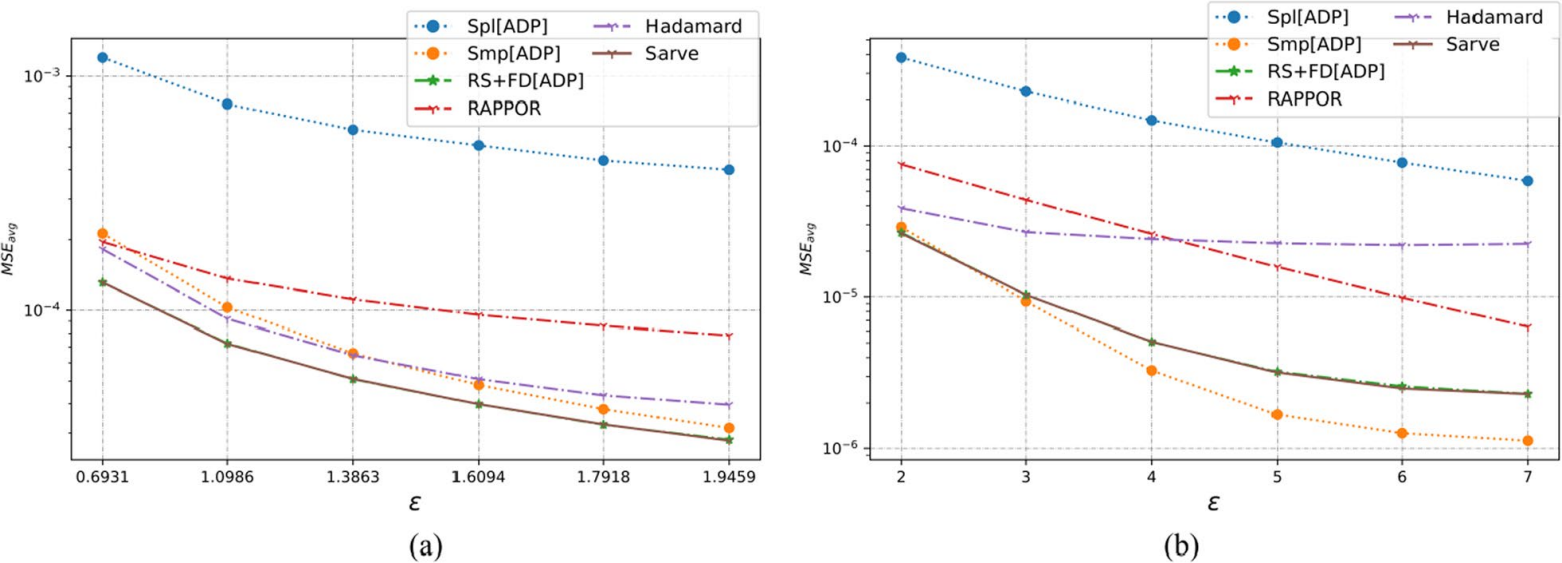
The MSE averaged over 100 runs for synthetic non-uniform data with N = 500,000, D = 20 privatized using different randomization techniques and fake data under **a** high privacy regime, experimental setup labeled ES_Syn_6 in Table 2; **b** general privacy regime, experimental setup labeled ES_Syn_12 in Table 2

The properties of the synthetic datasets used for benchmarking are summarized in Table 2. The algorithms were tested for uniform as well as non-uniform distributions for a large number of observations and different dimensions. The MSE averaged over a hundred runs for synthetic datasets post-privatization by Spl[ADP], Smp[ADP], RS + FD[ADP], RAPPOR, Hadamard Response, and *Sarve* have been shown in Figs. 8, 9, 10, 11, 12, and 13 respectively.

The effects of different factors like the number of observations attribute counts and domain values had affected the performance of randomization algorithms.

#### For high privacy regime

The numbers of observations were 50 K and 500 K with dimensionalities that took the values five and ten for uniform distribution, ten and twenty for non-uniform distribution. It was observed that the increase in the number of observations from 50 to 500 K had an adverse effect on the performance of Hadamard Response but RAPPOR remained immune to it. The performance of RAPPOR solely used as a randomization mechanism performed better than Smp[ADP] for all of the test cases. Interestingly, for a non-uniform distribution dataset having 50 K observations and ten attributes, HR performed better than RAPPOR. In all of the test configurations, *Sarve* performed on par with RS + FD[ADP].

#### For general privacy regime

The performance of RAPPOR as the sole randomization mechanism in the case of datasets with ten attributes was found better than Smp[ADP]. The recorded MSE showed a stable trend. The change in the number of attributes affected the performance of HR more than RAPPOR. Lastly, *Sarve* performed on par with RS + FD[ADP] with the former giving lower MSE for specific values of ε (Tables 6, 7).

The graphs were plotted for the MSE averaged over a hundred runs. For a clearer benchmarking between the existing solution and the proposed work, the lowest MSE reported by the methods has been summarized in Tables 3, 4 and 5.

## Conclusion

In this paper, the authors propose a novel frequency oracle termed *Sarve* for privacy-aware frequency estimation of categorical attributes of multi-user records. The privatization provided by *Sarve* utilized RAPPOR for randomization in addition to fake data. Existing research by Arcolezi et al. had used an adaptive combination of General Randomized Response and Optimal Unary Coding with fake data to prove that such mechanisms are well-suited for frequency oracles.

The use of a transformation-based method like Hadamard Response was found to perform on par with the existing work. The benefits offered by Hadamard Response include lower communication costs and therefore it emerged as a worthy alternative. Additionally, the implementation of *Sarve* tested the application of a hash-based method like RAPPOR. It was found that RAPPOR performed better than GRR and OUE for specific privacy conditions. Therefore, an adaptive privatization algorithm was devised to employ GRR, OUE, or RAPPOR based on the variance values. The proposed algorithms were tested on real-world as well as synthetic datasets that varied over the number of observations, dimensionalities, and size of allowed domain values. The adaptive performance of *Sarve* was found to be on par with the solution by Arcolezi et al. and performed better for specific privacy scenarios.

There is vast potential in the development of frequency oracles since all of the cloud-hosted services and platforms collect user information. The RAPPOR method is already in use by Google's Chrome browser and many popular tools. Therefore, the ability to amplify the privacy offered by such techniques in combination with fake data is an exciting avenue. This research can be extended to reduce the uncertainty introduced through the incorporation of fake data. Additionally, several other encoding schemes such as OLE exist that can be enhanced to produce frequency oracles like *Sarve*.

## Data Availability

The datasets used for the experiments are freely available to researchers. The links to the data have been cited as references.
